# Cuprous Chloride Nanocubes Grown on Copper Foil for Pseudocapacitor Electrodes

**DOI:** 10.1007/s40820-014-0007-3

**Published:** 2014-09-19

**Authors:** Bosi Yin, Siwen Zhang, Xin Zheng, Fengyu Qu, Xiang Wu

**Affiliations:** Key Laboratory for Photonic and Electronic Bandgap Materials, Ministry of Education, Harbin Normal University, Harbin, 150025 People’s Republic of China

**Keywords:** Cuprous chloride, Nanocubes, Electrochemistry, Supercapacitor

## Abstract

In this paper, for the first time, we report the synthesis of nanoscale cuprous chloride (CuCl) cubic structure by a facile hydrothermal route. A possible mechanism for the growth of those nanostructures is proposed based on the experimental results. It is discovered that the existence of HCl could affect the surface of CuCl nanocubes. This unique cube-like nanostructure with rough surface significantly enhances the electroactive surface areas of CuCl, leading to a high special capacitance of 376 mF cm^−2^ at the current density of 1.0 mA cm^−2^. There is still a good reversibility with cycling efficiency of 88.8 % after 2,000 cycles, demonstrating its excellent long-term cycling stability and might be the promising candidates as the excellent electrode material.

## Introduction

 The increasing demand for energy in the 21st century has triggered tremendous research efforts for energy storage and conversion from clean and renewable energy sources [1–7]. The supercapacitors represent an emerging class of energy storage devices that have attracted increasing attention because of a number of unique features including high power density, operating safety, environment benignity, fast charging/discharging rate, and long-term cycle stability [8, 9]. On the basis of the energy storage mechanism, the supercapacitors are classified into two types: electric double layer capacitors (EDLC) and pseudocapacitors. For EDLC, carbon materials (activated carbon, carbon nanotube, and graphene) are used as the electrode material. The charge storage is done by ion adsorption/desorption at the electrode/electrolyte interface [10–16]. In the case of pseudocapacitors, transition metal oxides and conducting polymers are used as the electrode material. The electrochromic behavior of transition metal oxides can store charges for long period without appreciable leakage [17], which makes them suitable for pseudocapacitor applications.

In the last decade, supercapacitor technology has undergone an increasing development owing to the discovery of new electrode materials, especially metal oxide nanomaterials, and the design of new hierarchical nanostructures [18–23]. For example, Wu et al. synthesized hierarchical SnO_2_ nanostructures assembled by many ultrathin nanosheets. They thought that their excellent supercapacitor performances could be ascribed to their unique morphology and the fast ion and electron transfer characteristics [24]. Gu et al. prepared WO_3_ nanowires and investigated their electrochemical performances [25]. Mai’s group reported ultra-long hierarchical vanadium oxide nanowires by electrospinning, which exhibit much higher capacity in lithium ion batteries [26]. However, most studies are currently focused on metal oxide and graphene composite materials.

CuCl is an I-VII semiconductor with a direct band gap of ~3.4 eV. It has a large exciton binding energy (~190 meV), which suggests the possibility of the fabrication of exciton-based blue/UV optoelectronic devices [27]. CuCl is widely used as a catalyst in organic synthetic industry. In this paper, for the first time, we prepared CuCl nanocubes by a facile hydrothermal route. The prepared CuCl products were used for the fabrication of the supercapacitor, and the results demonstrating the prepared nanomaterials represent the outstanding rate capability and high reversibility with little capacitance loss.

## Experimental

All the chemicals were of analytic grade and used as received without further purification. In a typical procedure, Cu substrate was cut into 1 × 1 cm^2^ pieces and immersed into acetone solution, ultrasonically cleaned for 30 min, and rinsed with deionized water. 5.5 mmol CuCl_2_ powder was dissolved into 20 mL deionized water with constant stirring. Another 20 mL deionized water was added into 8 ml concentrated HCl (its density is 1.18 g cm^−3^) in a 50 mL glass beaker. The mixed solution was then put into a 100 ml sealed Teflon-lined autoclave, followed by hydrothermal reaction at 140 °C for 12 h. Afterward, the autoclave was naturally cooled to room temperature. The substrate was taken out, rinsed with copious deionized water, and dried at 100 °C for 12 h in air.

The detailed morphologies of the samples were characterized by scanning electron microscope (SEM, Hitachi-4800). The chemical and elemental compositions of the prepared products were verified by energy dispersive spectroscopy (EDS), attached with SEM. The crystallinity of the prepared nanocubes was examined by X-ray diffractometer (XRD, Rigaku Dmax-2600/pc, Cu K radiation, *λ* = 0.1542 nm, 40 kV, 150 mA).

Electrochemical characteristics of the as-obtained products were studied on an CHI660 electrochemical work station (Chenhua, Shanghai) using cyclic voltammetry (CV) and electrochemical impedance test by configuring the samples into a three-electrode cell, where the substrate was used as the working electrode, Pt foil as the counter electrode, and an Ag/AgCl electrode as the reference electrode. The electrolyte used was 1 M Na_2_SO_4_ aqueous solution at room temperature. The electrochemical properties and capacitive behavior of the supercapacitor electrodes were evaluated by CV and galvanostatic discharge. The specific capacitance, *C* (mF cm^−2^), of the electrode material was calculated from the galvanostatic discharge according to the following equation:

$$C=I\times \;\Delta t/\left(\Delta V\times S\right)$$

where *I* is the discharge current (A), Δ*t* is the discharge time (s), Δ*V* is the voltage change (V) excluding IR drop in the discharge process, and *S* is the geometrical area of the electrode. The electrochemical impedance spectroscopy (EIS) measurements were performed by applying an AC voltage with 5 mV amplitude in a frequency range from 0.01 Hz to 100 kHz.

## Results and Discussion

**Fig. 1:**
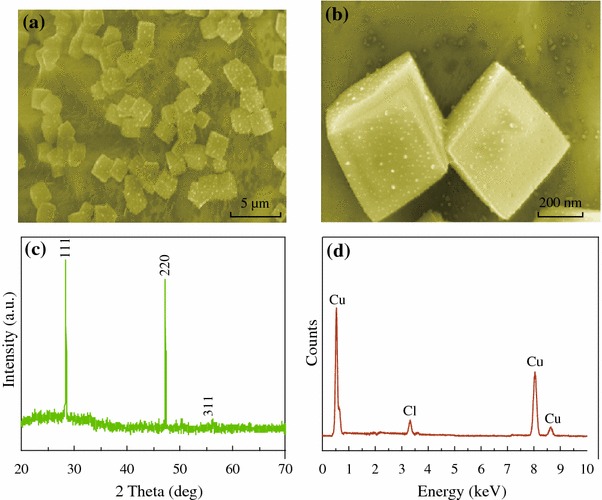
**a**, **b** SEM images of the as-synthesized CuCl nanocubes at different magnifications. **c** Typical XRD pattern of the as-synthesized CuCl products. **d** EDS spectra of the as-synthesized CuCl products

The general morphologies of the prepared CuCl products were investigated by SEM, and the results are demonstrated in Fig. 1a, b. As confirmed by the SEM observations, the prepared products possess well-defined and uniform cubic shapes. High magnification SEM image of individual CuCl nanocubes shows a rough surface. Figure 1c shows the typical XRD pattern of the as-prepared CuCl nanocubes. All the sharp peaks are in accordance with those of CuCl powder (JCPDS no. 06-0344). No peaks of other phases were detected, indicating the high purity of the as-synthesized product. Figure 1d shows an EDX spectrum. From this analysis, it was concluded that the cubes consist of about 80 % copper and 20 % chlorine.

**Fig. 2:**
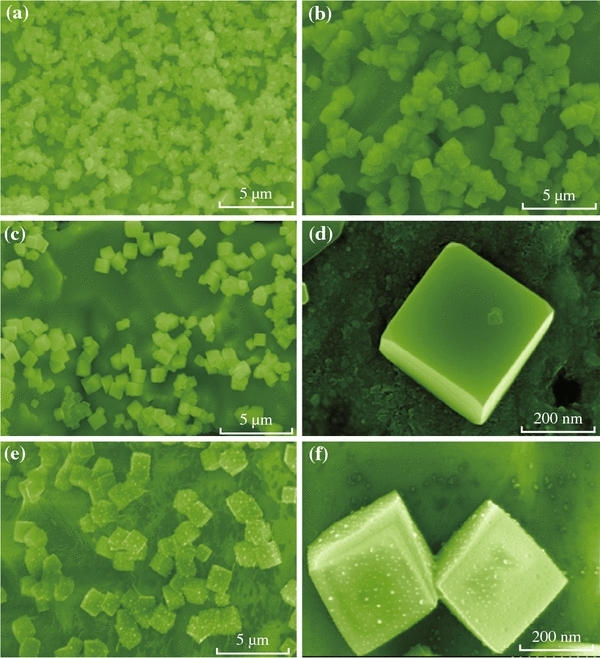
**a**–**f** Growth control of the product morphology. SEM images of the products at different times and different magnifications. **a** 6 h, **b** 8 h, **c** 10 h, **d** high magnification SEM image at 10 h, **e** 12 h, **f** high magnification SEM image at 12 h

**Fig. 3:**
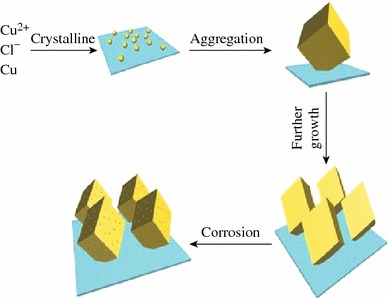
A growth schematic for the as-synthesized CuCl nanostructures

To further investigate the growth mechanism, controlled experiments were conducted by varying the reaction time. Figure 2a, b, c, d, e and f show time-dependent SEM images. For the prepared CuCl nanocubes, a possible growth mechanism can be proposed, as shown in Fig. 3. The following chemical reactions might occur [28]:

1 $${\text{Cu}}^{2+}+{\;\text{2Cl}}^{-}+\;\text{Cu}\;=\;\text{2CuCl}↓$$

2 $$\text{CuCl}+n{\text{Cl}}^{-}+m{\text{H}}_{2}\text{O}={\left[{\text{CuCl}}_{n}{\left({\text{H}}_{2}\text{O}\right)}_{m}\right]}_{}^{1-n}\left(n=0-4,m=0-6\right)$$

At first, a large number of tiny primary CuCl nanocrystals were formed due to the reaction (1). Crystal growth mechanisms of “self-assembly” and “oriented attachment” were suggested to dominate the growth process of CuCl nanocubes. The oriented attachment mechanism describes spontaneous self-organization of adjacent particles, and they share a common crystallographic orientation, followed by the joining of these particles at a planar interface. The process is particularly relevant in the nanocrystalline regime, where bonding between the particles reduces overall energy by removing surface energy associated with unsatisfied bonds [29]. In the reaction, Cu was used as the substrate, which can guide self-assembling growth of CuCl in aqueous solution without any surfactants and stabilizers. Then the “oriented attachment” can guide the oriented growth of the nanoparticles. As shown in Fig. 3, a supersaturated solution with plenty of CuCl small crystals was formed by adding Cu resource. Because of high surface energy and thermodynamics instability, CuCl nanoparticles grown on the surface of Cu substrate must decrease surface energy. The crystal growth prefers the orientation which maintains the minimum surface energy. Therefore, surface energy is substantially reduced when the neighboring nanocubes are grown. With the crystal growth continuing, each nanoparticle in the aggregates has its own orientation and acts as a nucleus for further growth. At the initial stage (about 140 °C for 10 h) (Fig. 2c, d), the surfaces of the nanocubes were smooth. As the growth duration increases, the smallest amount of CuCl reacts with H_2_O and Cl^−^ provided by HCl, and copper(I) chloride complexes have been carried out according to the reaction (2), and the surfaces of nanocubes are very rough [29].

**Fig. 4:**
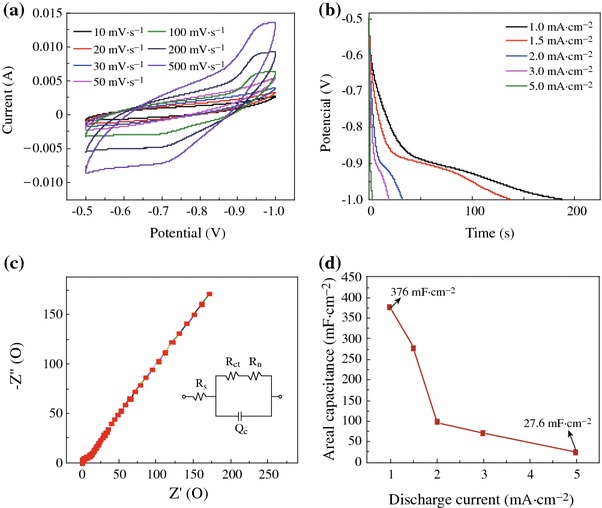
**a** CV curves at scan rates between 10 and 500 mV s^−1^; **b** discharge curves at current densities ranging from 1 to 5 mA cm^−2^; **c** impedance plots of CuCl electrode; **d** current density dependence of the areal capacitance

**Fig. 5:**
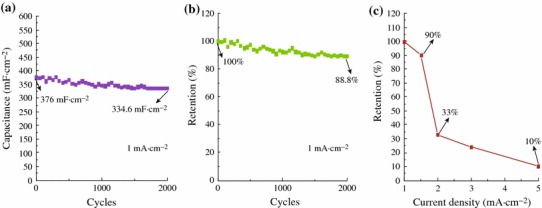
**a** Cycling performance at current density of 1 mA cm^−2^; **b** cycle life of CuCl electrode; **c** capacitance retention ratio as a function of discharge current densities

To highlight the merits of the obtained unique CuCl architectures and further explore their potential applications in the supercapacitors, the electrochemical performances of CuCl nanocubes as the integrated electrode were evaluated in three-electrode configuration with 1 M Na_2_SO_4_ aqueous solution as the electrolyte. CV curves of the working electrode collected at various scan rates ranging from 10 to 500 mV s^−1^ are shown in Fig. 4a. It shows that with the increase of the scan rate, the area enclosed by the CV curves increased, as the redox current increased. A galvanostatic discharging test was also performed with different current densities: 1.0, 1.5, 2.0, 3.0, and 5.0 mA cm^−2^, as shown in Fig. 4b. The linear voltage versus time profiles and a quick *I*–*V* response suggest that the CuCl nanocubes are good electrode materials in pseudocapacitors. EIS was applied to investigate the electrical conductivity and ion transfer of the supercapacitor cells. Figure 4c displays the Nyquist plots of CuCl product. The EIS data can be fitted by an equivalent circuit as shown by the inset in Fig. 4c. First, the intercept on the real axis in the high-frequency range provides the equivalent series resistance (ESR) (*R*_s_), which includes the inherent resistances of the electroactive material, the bulk resistance of electrolyte, and the contact resistance at the interface between the electrolyte and electrode. Its range also corresponds to the charge-transfer resistance caused by the Faradic reaction, which was correlated with the intercalation and deintercalation of ion. The charge transfer resistance (*R*_ct_), which results from diffusion of electrons, can be calculated from the diameter of the semicircle in the high-frequency range. The Warburg resistance (*R*_w_), which describes the diffusion of redox species in the electrolyte, can be reflected from the slope of the EIS curve in the low-frequency range [30]. Its range corresponds to the diffusion-limited mechanism, which confirms the main pseudocapacitive behavior. *Q*_c_ represents the constant phase element accounting for a double-layer capacitance [31]. The intercept of the Nyquist curve on the real axis (*R*_s_) manifests the good conductivity and the very low internal resistance of CuCl electrode, and consistent interfacial contact between CuCl and the Cu substrates. The enhanced electrochemical performance could be ascribed to the following structural features. First, separate cube-like structure leads to large open spaces to facilitate the electrolyte penetration and shorten the diffusion paths for both electrons and ions, resulting in reduced internal resistance. Second, Cu substrate can provide fast electronic transfer channels to improve the electrochemical performance. Moreover, Cl^−^ from electrolyte can successively induce CuCl and then form combination of CuCl–Cl together with the occurrence of electrons migration through the bond to access the underlying copper substrate [29]. The calculated capacitances as a function of discharge current densities are plotted in Fig. 4d. Impressively, CuCl electrode delivers special capacitance of 376, 276, 99, 72, and 27.6 mF cm^−2^ at current densities of 1.0, 1.5, 2.0, 3.0, and 5.0 mA cm^−2^, respectively. The enhanced electrochemical performance could be attributed to the following structural features. First, CuCl can absorb electrolyte anions (Cl^+^) on the electrode surface from electrolyte: (CuCl) surface + Cl^−^ + e^−^ ←→ (CuCl–Cl^−^) surface [27], providing more charge storage. Therefore, Cl^−^ from the electrolyte is fully utilized in CuCl electrode. Figure 5a, b, c demonstrates the cycling performance of the device up to 2,000 cycles at the current density of 1.0 mA cm^−2^. An areal capacitance of 376 mF cm^−2^ and a good reversibility with cycling efficiency of 88.8 % after 2,000 cycles are shown in Fig. 5b. Almost no obvious specific capacitance loss was observed, indicating its excellent long-term cycling stability. As seen in Fig. 5c, when the current density increases to 5 mA cm^−2^, the specific capacitance is 27.6 mF cm^−2^, maintaining 10 % of that at the current density of 1 mA cm^−2^.

## Conclusion

In summary, a mild hydrothermal method was used to fabricate CuCl nanocubes. The as-prepared product possesses a specific capacitance of 376 mF cm^−2^ at the current density of 1 mA cm^−2^ and a good reversibility with cycling efficiency of 88.8 % after 2,000 cycles. The synthesized CuCl nanocubes may have potential applications in energy storage and other electrochemical devices.
